# Characteristics and potential quality indicators for evaluating pre-travel consultations in Japan hospitals: the Japan Pretravel consultation registry (J-PRECOR)

**DOI:** 10.1186/s40794-021-00160-4

**Published:** 2022-02-01

**Authors:** Kei Yamamoto, Yusuke Asai, Issaku Nakatani, Kenichi Hayashi, Hidenori Nakagawa, Koh Shinohara, Shinichiro Kanai, Michitsugu Shimatani, Masaya Yamato, Nobuyuki Shimono, Tsuyoshi Kitaura, Nobuhiro Komiya, Atsushi Nagasaka, Takahiro Mikawa, Akihiro Manabe, Takashi Matono, Yoshihiro Yamamoto, Taku Ogawa, Satoshi Kutsuna, Norio Ohmagari

**Affiliations:** 1 Disease Control and Prevention Center/Travel Clinic, National Center for Global Health and Medicine, 1-21-1 Toyama, Shinjuku-ku, Tokyo, 162-8655 Japan; 2 Travel Clinic, Nara Seibu Hospital, Nara, Japan; 3 Department of Infectious Diseases, Kenwakai Otemachi Hospital, Kitakyushu, Japan; 4 Department of Infectious Diseases, Osaka City General Hospital, Osaka, Japan; 5 Department of Infectious Diseases, Kyoto City Hospital, Kyoto, Japan; 6 Department of Infection Control, Shinshu University Hospital, Matsumoto, Japan; 7 Department of Infectious Diseases, Hamamatsu Medical Center, Hamamatsu, Japan; 8 Department of General Medicine and Infectious Diseases, Travel Clinic, Rinku General Medical Center, Izumisano, Japan; 9 Center for the Study of Global Infection, Kyushu University Hospital, Fukuoka, Japan; 10 Center for Infectious Disease, Tottori University Hospital, Yonago, Japan; 11 Department of Infectious Diseases, Japanese Red Cross Society Wakayama Medical Center, Wakayama, Japan; 12 Department of Infectious Diseases, Sapporo City General Hospital, Sapporo, Japan; 13 Department of General Medicine and Infectious Diseases, Yamanashi Prefectural Central Hospital, Kofu, Japan; 14 Department of Clinical Laboratory Medicine, Fukuyama City Hospital, Fukuyama, Japan; 15 Department of Infectious Diseases, Aso Iizuka Hospital, Iizuka, Japan; 16 Department of Clinical Infectious Diseases, Toyama University Hospital, Toyama, Japan; 17 Center for Infectious Diseases, Nara Medical University Hospital, Kashihara, Japan

**Keywords:** Japan, Quality indicators, Health care, Pre-travel consultation, Registries

## Abstract

**Background:**

Awareness of pre-travel consultations (PTCs) and prevention methods for overseas travel-related diseases, and the understanding of PTCs among Japanese travelers and medical professionals remains low in Japan. A multicenter registry was established to examine PTCs in Japan. This study assessed the PTC implementation rate and examined the indicators of PTCs that can be used as criteria for evaluating quality.

**Methods:**

Clients who presented for their PTCs at 17 facilities and were registered between February 1, 2018, and May 31, 2020, were included. Medical information was extracted retrospectively via a web-based system. Correlations between vaccination risk categories and advice/intervention proportions by the facility were evaluated using Spearman’s ordered phase relations (α = 0.05).

**Results:**

Of the 9700 eligible clients (median age, 32 years; 880 [9.1%] aged < 16 years and 549 [5.7%] aged ≥65 years), the most common travel duration was ≥181 days (35.8%); higher among younger clients. The most common reason for travel was business (40.5%); the US (1118 [11.5%]) and Asia (4008 [41.3%]) were the most common destinations and continents, respectively. The vaccine number (median three per person) increased after the PTCs except for the tetanus toxoid. Only 60.8% of the clients recommended for malaria prophylaxis received anti-malarial agents. The gross national income; the incidence of human rabies, typhoid fever, falciparum malaria; and dengue risk category were associated with the percentage of hepatitis-A vaccines; explaining rabies post-exposure prophylaxis, typhoid-fever vaccinations, malaria-prophylaxis prescriptions; and mosquito repellants, respectively.

**Conclusions:**

Although the characteristics of the travelers differed, the quality of the PTCs should be improved to address, for example, the lower rate of acceptance of malaria prophylaxis in Japan.

**Supplementary Information:**

The online version contains supplementary material available at 10.1186/s40794-021-00160-4.

## Background

Although the number of travel clinics registered with the Japanese Society of Travel and Health that provide pre-travel consultations (PTC) in Japan increased from 45 to 90 between 2011 and 2016, an airport survey showed that awareness of PTC among Japanese travelers remained low when compared to other countries [1–5]. Therefore, how PTC is implemented in Japan and the needs of travelers remain unclear. Hence, the Japan Pretravel Consultation Registry (J-PRECOR), a multicenter registry of general hospitals in Japan, which manages travel clinics, was established.

An objective of this registry is to ensure equivalence in the quality of PTC care across Japan by considering the criteria used for the evaluation of PTC quality. Although the quality of PTCs has been evaluated using questionnaire surveys or prospective observational studies among health care providers in other countries [6–8], evaluation guidelines have not yet been established. Therefore, this study evaluated the variations in PTC implementation rates according to specified indicators (the risk of food-borne infectious diseases, mosquito-borne diseases, and rabies) among the facilities, based on real-world data collected from multiple institutes, and examined the indicators of PTCs that could be used as criteria for evaluating quality.

## Methods

In this multicenter retrospective study, clients’ data were extracted from the hospitals’ registry from February 1, 2018, to May 31, 2020. Clients who only underwent health check-ups for travels abroad or were not planning to travel abroad were excluded. Furthermore, clients with missing country, date, and purpose of travel were also excluded. PTCs were treated as separate if the purpose or countries of travel was different. The clients’ data were extracted retrospectively from the clinical records when the schedule of immunizations and/or prescriptions for the clients was determined. Four co-operating hospitals were registered at the beginning of the study, while overall, 17 hospitals had registered clients during the study period. Of these 17, four were yellow fever vaccine (YFV)-capable hospitals, and 11 administered unapproved vaccines in Japan (Supplementary Table 1 in Additional file 1).

Demographic and medical data were extracted (Supplementary Material in Additional file 1), and the approved and unapproved vaccines in Japan as of March 2020 were aggregated (Supplementary Table 2 in Additional file 1).

The travel duration was categorized into 1–7, 8–14, 15–28, 29–181, and > 181 days. Country income was categorized into low, lower-middle, upper-middle, and high according to the gross national income (GNI) as published by the World Bank [9]. The following were classified according to the number of deaths that were due to rabies (per 100,000 population) [10], typhoid fever (per 1000 population) [11], and confirmed *Plasmodium falciparum* malaria (per 1000 population) [12]. The dengue fever risk categories (“Frequent/continuous,” “Sporadic/uncertain,” “Risk variations based on region,” and “No/unknown risk”) were defined as reductions in the risk levels, in that order [13]. For travel to multiple countries, the GNI was calculated and classified according to the country with the lowest income, while the risk of diseases was calculated and classified according to the country with the highest risk. To evaluate the quality of the PTCs, the percentages of interventions and advice implemented were calculated according to the categories (Supplementary Material Methods in Additional file 1) and the facilities. Data with no more than five applicable cases in each category were excluded from the figure without calculating the percentage. We also conducted subgroup analyses of the vaccination and prophylactic medication prescription rates, stratified by the duration and purpose of travel.

The protocol was approved by the Institutional Review Board (IRB) of the National Center for Global Health and Medicine (NCGM) (NCGM-G-002347-01) and the IRBs/Ethical Committees of the other cooperating facilities. The study information was presented in a poster and on the Web to allow the clients to opt out.

### Statistical analysis

The discrete data were expressed as numbers (percentages), while the continuous data were expressed as medians (interquartile ranges [IQR]). The correlations between the vaccination risk categories and the advice/intervention proportions by the facility were evaluated using Spearman’s ordered phase relations (α = 0.05). All the statistical analyses were performed using IBM SPSS Statistics for Windows, version 26.0 (IBM Corp., Armonk, N.Y., USA).

## Results

### Characteristics of the travelers (Table 1)

Of the 9746 registered clients, 46 with missing values were excluded, leaving 9700 (Supplemental Table 3 in Additional file 1). The overall median age was 32 [21–45] years; 880 (9.1%) and 549 (5.7%) clients were aged 0–15 and ≥ 65 years, respectively (Table 1). The duration of travel was known in 9190 (94.7%) clients, and the most common duration was > 181 days (35.8%), with the most common reason for travel overall being business (3930, 40.5%). By country, the US (1118 [11.5%]) was the most common destination, followed by Brazil (1001 [10.3%]), while Asia was the most common continent with 4008 (41.3%) clients.

**Table 1:** Characteristics of the participants by age group

	All	Age 0 to 15 years	Age 16 to 64 years	Age 65 years or over
Number of clients	9700	880	8271	549
Male (%)	5806 (59.9)	436 (49.5)	5042 (61)	328 (59.7)
Female (%)	3894 (40.1)	444 (50.5)	3229 (39)	221 (40.3)
Age, median, years [IQR]	32 [21,45]	6 [3,11]	32 [23,43]	69 [67,72]
Days from first consultation to travel, median, days [IQR]	33 [17,60]	50 [25,96]	32 [16,57]	35 [20,60]
Immunization record (%)	4876 (50.3)	666 (75.7)	4113 (49.7)	97 (17.7)
Request for vaccine (%)	7793 (80.3)	657 (74.7)	6726 (81.3)	410 (74.7)
Travel period (%)				
less than 7 days	675 (7.3)	14 (1.7)	622 (7.9)	39 (7.3)
7–13 days	2272 (24.7)	75 (9.1)	1936 (24.7)	261 (48.8)
14–27 days	1468 (16)	55 (6.6)	1270 (16.2)	143 (26.7)
28–55 days	811 (8.8)	35 (4.2)	732 (9.4)	44 (8.2)
56–181 days	674 (7.3)	29 (3.5)	616 (7.9)	29 (5.4)
more than 181 days	3290 (35.8)	620 (74.9)	2651 (33.9)	19 (3.6)
Travel purpose (%)				
Group tourism	640 (6.6)	26 (3.0)	413 (5.0)	201 (36.6)
Individual tourism	1910 (19.7)	70 (8.0)	1681 (20.3)	159 (29.0)
Business	3930 (40.5)	10 (1.1)	3790 (45.8)	130 (23.7)
Moving with family	1198 (12.4)	588 (66.8)	602 (7.3)	8 (1.5)
Migration	26 (0.3)	7 (0.8)	17 (0.2)	2 (0.4)
Study	1330 (13.7)	127 (14.4)	1201 (14.5)	2 (0.4)
Volunteer work	472 (4.9)	14 (1.6)	441 (5.3)	17 (3.1)
Visiting friends/relatives	132 (1.4)	42 (4.8)	76 (0.9)	14 (2.6)
Others	214 (2.2)	15 (1.7)	167 (2.0)	32 (5.8)
Most visited countries (%)				
First	USA 1118 (11.5)	USA 173 (19.7)	USA 916 (11.1)	Brazil 114 (20.8)
Second	Brazil 1001 (10.3)	China 101 (11.5)	Brazil 812 (9.8)	Kenya 85 (15.5)
Third	China 769 (7.9)	Brazil 75 (8.5)	China 662 (8.0)	Tanzania 53 (9.7)
Fourth	Kenya 750 (7.7)	Thailand 70 (8.0)	India 647 (7.8)	South Africa 49 (8.9)
Fifth	India 696 (7.2)	Indonesia 41 (4.7)	Kenya 647 (7.8)	Peru 39 (7.1)
Visit more than one country (%)	1666 (17.2)	20 (2.3)	1471 (17.8)	175 (31.9)
Visit low or lower-middle income countries included (%)	5067 (52.2)	252 (28.6)	4502 (54.4)	313 (57)

IQR, interquartile range; USA, United States of America

The younger the age, the more likely they were to travel for longer than 181 days, with 74.9% of those aged 15 or younger traveling for longer periods. For those aged 15 and under, the most common reason for travel was to accompany family members (66.8%), followed by study abroad and school events (14.4%). However, more than half of the elderly (65 years old and over) traveled for two weeks or less, with the purpose of their trip being mainly sightseeing (65.6%), and more of them traveling in groups on package tours (36.6%) than those in any other age group.

Except for the YFV, the most common vaccines requested were against hepatitis A, rabies, tetanus, and hepatitis B (Table 2). Vaccines were required in 7793 clients (80.3%). Those traveling outside Asia, Africa, and Latin America, made more requests for vaccines against measles, rubella, meningococcal, and Tdap than those planning to travel to these regions. YFV was requested by 3014 clients. The proportion of YFV requests in those aged ≥65 years was higher (52.3%) than in those aged < 16 years (14.4%) and 16–64 years (31.4%). Altitude sickness and malaria prophylaxes were the most requested by travelers to Latin America (77.2%) and Africa (72.4%) (Table 2).

**Table 2:** Differences between the interventions that the participants wanted to use and the interventions that they actually used after the travel consultations

	Vaccines and prescriptions that the participants wanted to use themselves	Vaccines and prescriptions actually given after pre-travel consultation	*Percentage difference	**Change ratio	***P value
Hepatitis A vaccine	3946	5655	17.6	1.43	< 0.001
Hepatitis B vaccine	2562	2961	4.1	1.16	< 0.001
Rabies vaccine	2804	3209	4.2	1.14	< 0.001
Vaccines containing tetanus toxoid	3017	4625	16.6	1.53	< 0.001
Tdap	151	597	4.6	3.95	< 0.001
DTaP	471	2388	19.8	5.07	< 0.001
Typhoid fever vaccine	1513	2468	9.8	1.63	< 0.001
Japanese encephalitis vaccine	1231	1745	5.3	1.42	< 0.001
Meningococcal ACWY vaccine	463	772	3.2	1.67	< 0.001
Meningococcal B vaccine	8	32	0.2	4.00	< 0.001
Vaccines containing measles	772	2012	12.8	2.61	< 0.001
Vaccines containing rubella	682	2006	13.6	2.94	< 0.001
Yellow fever vaccine	3014	3559	5.6	1.18	< 0.001
Prophylaxis for acute altitude sickness	338	370	0.3	1.09	< 0.05
Prophylaxis for malaria	1146	1252	1.12	1.10	< 0.001

*Percentage difference: percentage after pretravel consultations minus that before pretravel consultations (after - before)

**Change ratio: Ratio of the number of cases after pretravel consultations to that before pretravel consultations (after/ before)

***Compared using the McNemar test

### Interventions

Following the PTC, the median number of and most common vaccines planned were three (IQR, 1–4) per person. Several travelers to Asia were vaccinated against hepatitis A, hepatitis B, rabies, and typhoid (Supplementary Table 4 in Additional file 1). Of the 29,082 planned vaccines, 24.5% were unapproved in Japan. Of the unapproved vaccines, the most frequently used vaccines were the adjuvant-containing hepatitis A, typhoid fever, and rabies vaccines.

Overall, the number of planned vaccines after PTC increased compared to the required vaccines before PTC (*P* < 0.05), especially the vaccines containing measles and rubella, and diphtheria, tetanus, and pertussis (Table 2 and Supplementary Table 4 in Additional file 1). The number of planned meningococcal vaccine recipients was small; however, this showed a marked increase after consultation (Table 2). The numbers of rabies, hepatitis B, yellow fever, and Japanese encephalitis vaccines were generally similar between the planned and requested numbers. The YFV number of consultations was higher among the ≥65-year-olds than among the < 65-year-olds (60.1% vs. 35.3%, *P* < 0.001). The percentage of prescriptions for altitude sickness prophylaxis did not change significantly after the consultation. For malaria prophylaxis, there was a slight increase in those planning travels to Africa, and conversely, a decrease in those traveling to other regions.

Malaria prophylaxis or emergency standby treatment was recommended in 22.5% (2180/9700) of clients and in 34.8% (1821/5226) of those traveling for < 56 days; and especially in those who planned to travel to the African region (68.8%, 1429/2078). Among those traveling for < 56 days, two clients each had unknown prescription status and planned emergency standby treatment. Besides these, only 60.8% of those recommended for malaria prophylaxis received the prescriptions. The most common destination countries for which malaria prophylaxis was prescribed were Kenya, Tanzania, Uganda, and Ghana. However, even in countries with A high malaria risk (over 10 confirmed cases per 1000 populations), the prescription rate for those who received prophylaxis recommendations varied from 42.1 to 84.2% (Supplementary Table 5 in Additional file 1).

The most common advice that was given was for the use of rabies post-exposure prophylaxis (PEP), mosquito repellent use, and dietary precautions, all of which were common among travelers to Asia and Africa (Table 3).

**Table 3:** Advice given during consultations by region of travel

	All	Asia	Africa	South America	Others	Multi
N (%)	9700	4008	2593	1809	1660	1670
Explanation of post-exposure prophylaxis for rabies	6436 (66.4)	3115 (77.7)	1784 (68.8)	1207 (66.7)	597 (36)	1097 (65.7)
How to use mosquito repellents	6486 (66.9)	2841 (70.9)	2092 (80.7)	1394 (77.1)	457 (27.5)	1248 (74.7)
Explanation of the risk of leptospirosis and/or schistosomiasis due to freshwater exposure	2901 (29.9)	1514 (37.8)	740 (28.5)	454 (25.1)	314 (18.9)	476 (28.5)
Explanation of dietary habits to avoid foodborne diseases	5780 (59.6)	2842 (70.9)	1592 (61.4)	1071 (59.2)	507 (30.5)	998 (59.8)
Avoiding traffic accidents	3286 (33.9)	1817 (45.3)	626 (24.1)	440 (24.3)	501 (30.2)	482 (28.9)
Preventive actions for acute mountain sickness	768 (7.9)	304 (7.6)	121 (4.7)	256 (14.2)	134 (8.1)	185 (11.1)
Discussing the risks and prevention of sexually transmitted diseases	809 (8.3)	321 (8)	280 (10.8)	179 (9.9)	85 (5.1)	198 (11.9)
Taking overseas travel accident insurance	3359 (34.6)	1436 (35.8)	954 (36.8)	640 (35.4)	456 (27.5)	596 (35.7)
Others	116 (1.2)	64 (1.6)	18 (0.7)	13 (0.7)	26 (1.6)	11 (0.7)

### Quality indicators

#### The GNI category, percentage of hepatitis a vaccination (HAV) planning, and dietary advice to prevent foodborne diseases (Fig. 1)

These vaccines and advice were correlated weakly with each of the GNI categories respectively (*ρ* = 0.37, *P* < 0.01; *ρ* = 0.41*, P* < 0.01). These vaccines and advice tended to be considered when people were traveling to low or upper-middle-income countries. The width of interquartile range for hepatitis A vaccine coverage was 35.8, 32.0, 17.7, and 50.2, in the high, upper-middle, lower-middle, and low GNI categories, respectively, while food advice was 44.8, 50.6, 55.1, and 50.0, respectively. Compared to the advice on HAV, there was a greater difference in advice about eating and drinking among the facilities. The subgroup analysis according to the purpose of travel showed that overall, the median vaccination rates for upper-middle and lower-middle GNI categories were 86.9 and 88.6%, respectively, for travel for business, which were higher than those for tourism (66.9 and 72.7%, respectively). Although the low GNI category, on the contrary, had a low median vaccination rate of 37.5%, there were only four facilities in this category, and the range was highly variable, ranging from 0 to 87.5% (Supplementary Fig. 1 in Additional file 2).

**Fig. 1:**
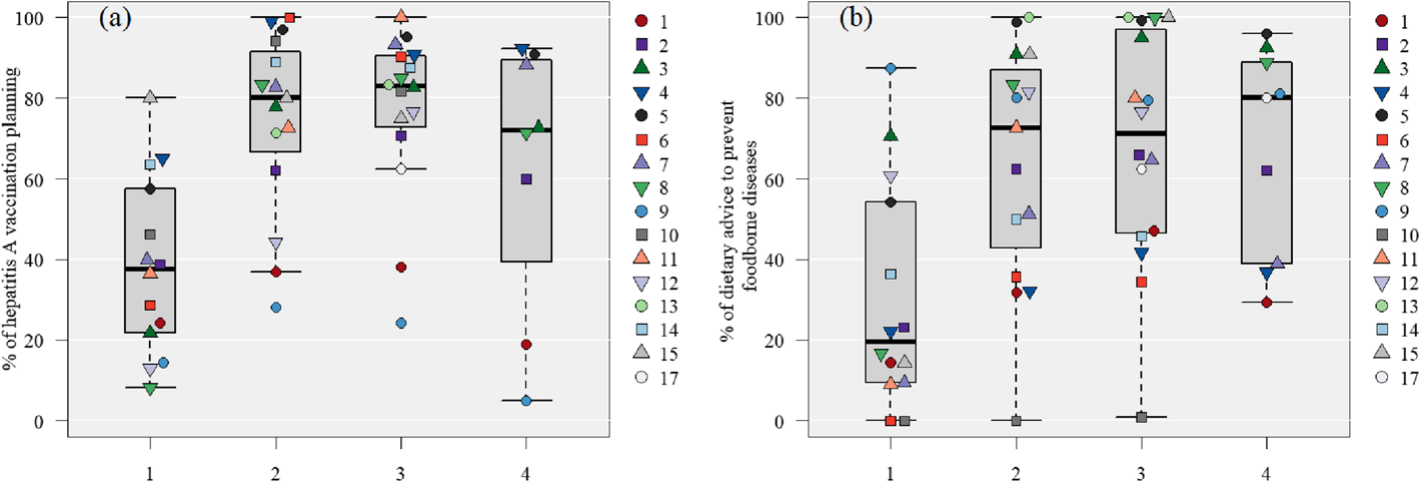
Country classification by income level and interventions implemented during pre-travel consultations by each collaborating hospital. The numbers in the legend correspond to the “Hospital number.”. **A**: The percentage of hepatitis A vaccine planning in clients without immunization histories of hepatitis A vaccines categorized by the gross national income (GNI). The risk categories, in order, from 1 to 4, are “high GNI: $12,536 or more”; “upper-middle GNI: $4,046 and $12,535”; “lower-middle GNI: $1,036 and $4,045”; and “low GNI: $1,035 or less.” Cases in which hepatitis A vaccine had been administered, with vaccination histories, were excluded. Of the 8204 patients included in the validation, data from 10 patients (one facility) were excluded because they could not be classified into a risk category. **B**: The advisory rate of dietary habits to avoid foodborne diseases categorized by the GNI. The risk categories, in order, from one to four, are “high GNI: $12,536 or more”; “upper-middle GNI: $4046 and $12,535”; “lower-middle GNI: $1036 and $4045”; and “low GNI: $1035 or less.” Of the 9658 patients included in the validation, data from 11 patients (one facility) were excluded because they could not be classified into a risk category. * Refer to the supplementary materials for the country names (ISO 3166-1 codes, Alpha-3 code) included

#### The risk category of rabies, the percentage of pre-exposure prophylaxis (PrEP) planning, and explaining post-exposure prophylaxis (PEP) (Fig. 2)

As the rabies incidence rate increased, the percentage of explaining PEP increased for those that planned to travel to high-risk rabies countries (*ρ* = 0.30, *P* < 0.01). However, the percentage of explaining PEP was quite low in some facilities, as with the other advice. Furthermore, the rate of PrEP planning was not related to the incidence rate (*P* > 0.05). There was a tendency for the implementation rate to decrease in most facilities in countries with a slightly high risk of human rabies (0.6–1.5 deaths per 100,000 population), including African countries (such as Kenya and Tanzania), which had relatively large numbers of visitors from Japan. When analyzing the PrEP implementation in long-term travel, ≥181 days, the clients planning long-term travel had an overall higher median vaccination coverage ranging from 37.6–92.1% across all categories, compared to only 11.8–43.1% for travel < 181 days (Supplementary Fig. 2 in Additional file 2).

**Fig. 2:**
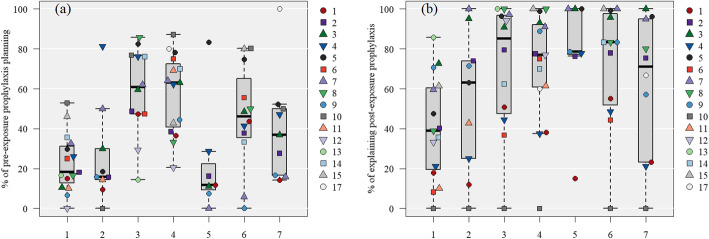
Risk classification of rabies and interventions implemented during pre-travel consultations by each collaborated hospital. The numbers in the legend correspond to the “Hospital number.”. **A**: Percentage of rabies pre-exposure prophylaxis (PrEP) planning in clients without history of completed PrEP categorized by the risk of rabies. The risk categories, in order, from one to seven, are death rates due to human rabies per capita (per 100,000 persons): “less than 0.0024”; “0.0024 to less than 0.038”; “0.038 to less than 0.19”; 0.19 to less than 0.6″; “0.6 to less than 1.5”; “1.5 to less than 3.0”; and “3.0 or more.” Cases in which rabies PrEP had been administered, with a vaccination history, were excluded. Of the 8803 patients included in the validation, data from 136 patients (five facilities) were excluded because they could not be classified into a risk category. **B**: The rate of explaining post-exposure prophylaxis categorized by the risk of rabies. Risk categories were defined in the same way as in (**C**). Of the 9618 patients included in the validation, data from 145 patients (five facilities) were excluded because they could not be classified into a risk category. * Refer to the supplementary materials for the country names (ISO 3166-1 codes, Alpha-3 code) included

#### The risk category of typhoid fever and the percentage of typhoid fever vaccination planning

**Fig. 3:**
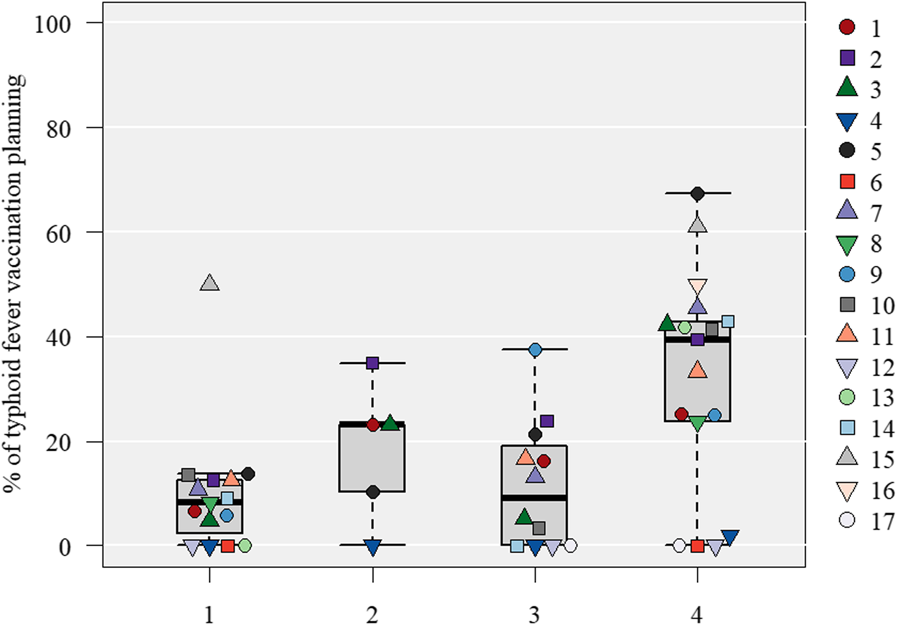
Risk classification of typhoid fever and vaccinations administered during pre-travel consultations by each collaborated hospital. The numbers in the legend correspond to the “Hospital number.” Percentage of typhoid fever vaccination planning in clients without immunization history of typhoid fever vaccine within three years, categorized by the risk of typhoid fever. The risk categories, in order, from one to four, are the incidence of typhoid fever per 100,000 persons: “less than 20”; “20 to less than 50”; “50 to less than 100”; and “100 or more.” Cases in which typhoid fever vaccine within three years had been administered, with vaccination histories, were excluded. Of the 9333 patients included in the validation, data from 26 patients (two facilities) were excluded because they could not be classified into a risk category. * Refer to the supplementary materials for the country names (ISO 3166-1 codes, Alpha-3 code) included

Both the percentage of typhoid fever vaccine planning and advice tended to increase in proportion to the incidence of typhoid fever (*Ρ* = 0.41, *p* < 0.01). Since the typhoid vaccine is unapproved in Japan, the vaccine planning rate was lower in facilities that did not handle unapproved vaccines. The analysis by the purpose of travel showed no difference in trends. However, in risk category 4, the median vaccination rates were 40.0 and 31.3% for travel for business and purposes other than for business and tourism, respectively, while the rate for travel for tourism was lower at 16.5% (Fig. 3 and Supplementary Fig. 3 in Additional file 2).

#### Recommendations for prevention of mosquito-borne diseases and implementation of mosquito control advice

**Fig. 4:**
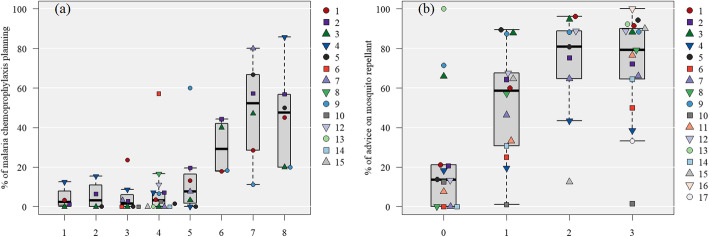
Risk classification of mosquito-borne diseases and interventions implemented during pre-travel consultations by each collaborated hospital. The numbers in the legend correspond to the “Hospital number.”. **A**: The prescription rate of malaria prophylaxis in clients traveling for < 56 days is categorized by the risk of falciparum malaria. The risk categories, in order, from one to eight, are the incidence of falciparum malaria per 1000 persons: “no risk”; “less than 0.1”; “0.1 to less than 1.0”; “1.0 to less than 10”; “10 to less than 50”; “50 to less than 100”; “100 to less than 250”; and “250 or more.” Of the 5124 patients included in the validation, data from 403 patients (eight facilities) were excluded because they could not be classified into a risk category. **B**: Implementation of mosquito control advice about mosquito repellant, categorized by the risk of dengue fever: The risk categories, in order, from zero to three, are classified according to the reference 12 dengue risk categories: “no or unknown risk”; “risk varies based on region”; “sporadic/uncertain”; and “frequent/continuous.”. * Refer to the supplementary materials for the country names (ISO 3166-1 codes, Alpha-3 code) included

For *P. falciparum* malaria, the higher the incidence in the destination country, the higher the rate of preventive medication prescription plans (*Ρ* = 0.66, *p* < 0.001). The percentage of advice on mosquito repellant use was not significantly higher for those traveling to high-risk countries according to the dengue fever risk category (*P* > 0.05). In the analysis by the purpose of travel, although the prescription rate was lower in the highest risk category 8, tourism purposes, there were no noticeable differences between travel purposes. Moreover, in this subgroup analysis, the number of facilities with a certain number (*n* ≥ 5) of travelers to high-risk category areas, was low (Fig. 4 and Supplementary Fig. 4 in Additional file 2).

#### The catch-up vaccination rate of measles-containing vaccines to the clients (with and without vaccination records) by age

**Fig. 5:**
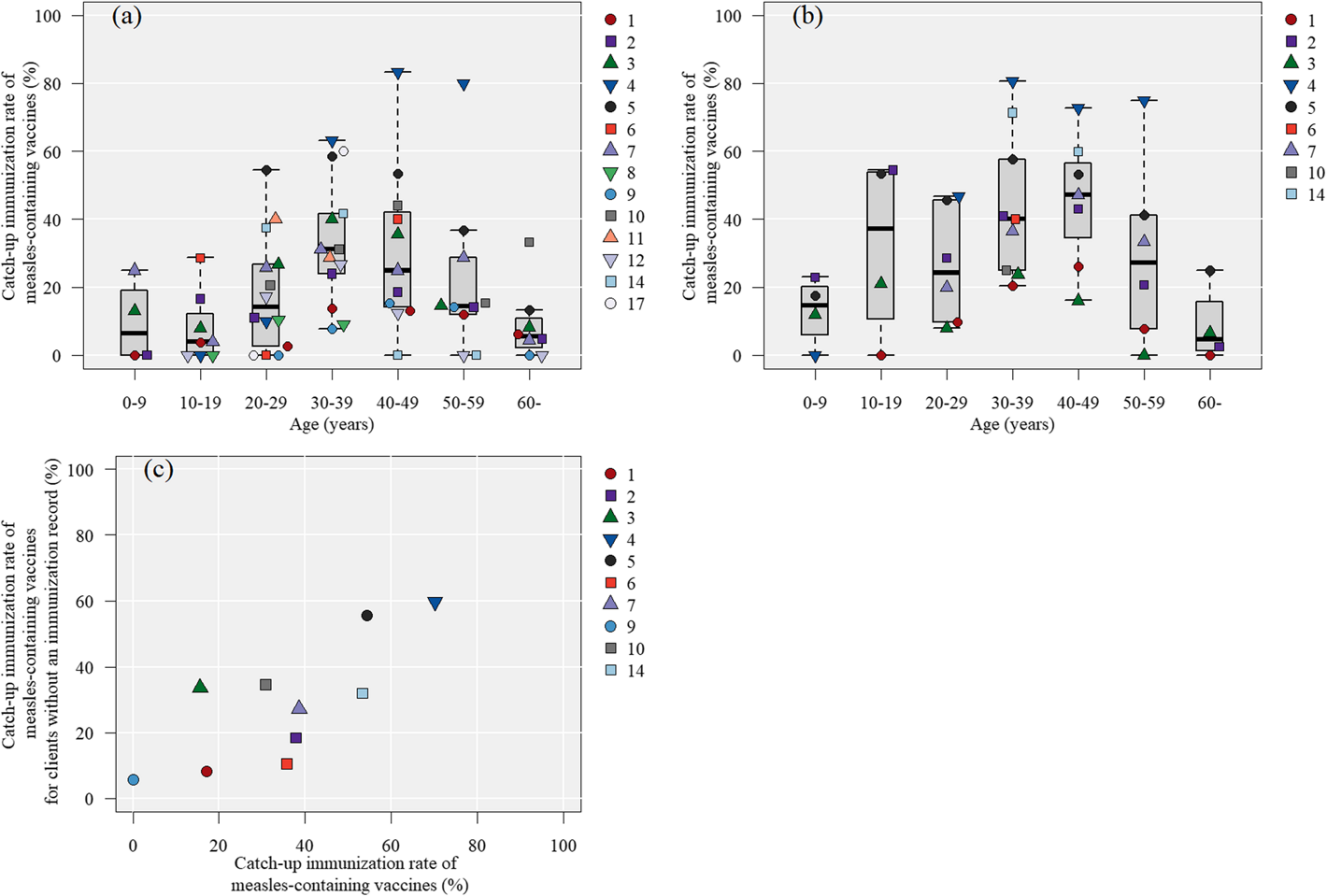
Catch-up immunization rate of measles-containing vaccines by age group in each facility. The numbers in the legend correspond to the “Hospital number.” Data with no more than five applicable cases in each category were excluded from the figure without calculating the percentage. **A**: Catch-up immunization rates of measles-containing vaccines by age group among participants without vaccination records (4643 patients were included in the validation). **B**: Catch-up immunization rates of measles-containing vaccines by age group among participants with vaccination records who have received none or one measles-containing vaccine (2551 patients included in the validation). **C**: Association between catch-up immunization rates among participants without vaccination records and subjects with vaccination records that require catch-up measles-containing vaccines

For those without immunization records, many facilities tended to immunize more clients in their 30s and 40s, with less natural immunity and who were likely to have been immunized once (Fig. 5A and B). For those with records, the catch-up immunization rate was relatively high among those in their teens compared to those in their 50s. However, regardless of vaccination histories, there were strong inter-institutional variations in the measles-containing vaccine coverage (Fig. 5C).

## Discussion

The characteristics of clients who presented themselves for PTC in Japan in this study showed that 35.8% were long-term travelers with a travel period of > 6 months. In PTCs in other countries, the clients who traveled for > 6 months were few, ranging from 3 to 6.5% [14, 15]. However, in Scotland and China, there have been reports that long-term travel is more common (22.7–78.2%) [16, 17]. According to the PTCs of other countries, although business was the most common reason for travel, the main purpose of travel was tourism (49.4 to 74.8%) [14, 15, 17–20]. Unlike these other countries, Japan showed a similar trend, although not as much as in the previously mentioned China report [16], in that there were several long-term travelers for business. Compared to previous European reports [15, 18, 19], the percentage of those who traveled to Africa was slightly lower, with most destination countries being in Asia. More than half of the clients traveled to low- to lower-middle-income countries, which was slightly lower than that reported in the US [14]. However, there was no difference in that many clients traveled to high-risk health problem countries. In an airport survey of mainly tourism Japanese travelers [2], the travel clinic consultation rate was very low at only 2%. This rate is clearly lower than the rates of PTCs in airport surveys conducted in other countries [3–5]. This is considered a major problem faced by Japanese travelers abroad. The age groups of the clients in this study were generally the same as those reported in the PTCs in other countries [14, 15, 18]. Although Japan had the highest percentage of people aged > 65 years, worldwide [21], the percentage of those aged > 65 years who received pre-departure counseling was approximately 6%, which was similar to the percentages in the US and Europe (4.6 to 9%) [14, 18]. These results were due to the small number of older adults who traveled abroad [22], rather than that they do not present for PTC. Most elderly clients who visited the clinic for consultation were traveling for tourism, most commonly to Brazil, Kenya, and Tanzania, and it was likely that their consultations were for YFV. Unlike a report from Greece [19], with a significantly low percentage of vaccination in clients aged ≥65 years against yellow fever, this study showed a high percentage for those who received YFV. This difference may be attributed to the vaccination system in Japan. The YFV facilities in Japan comprise 19 quarantine stations and medical institutions nationwide [23]. Hospitals 1, 2, 9, and 12 were among these 19 facilities (Supplementary Table 1 in Additional file 1). Because of the high incidence of serious adverse reactions in the elderly, vaccination at medical institutions is often recommended by the quarantine offices to avoid vaccination at the quarantine stations. Therefore, many older adults visit travel clinics for YFV vaccinations because of this recommendation, and it is assumed that the rate of YFV vaccination is high.

The acceptance of malaria chemoprophylaxis in recommended cases among travelers who planned to travel for < 2 months (60%) was lower than the acceptance rate in other countries (70.7 to 80.5%) [15, 18]. The airport survey also showed that only 20% of travelers to malaria high-risk countries received malaria chemoprophylaxis [24], suggesting the need for disease education and prevention awareness. However, there were two possible reasons for the low acceptance rate in this study. First, the number of facilities in Japan that can conduct YFV upon entry into yellow fever risk countries, where malaria prophylaxis is often required, is limited. It is assumed that a considerable number of clients come to the hospital after completing their PTCs at another hospital. This is also likely to be the reason for the low prescription rate of malaria prophylaxis in the malaria high-risk countries’ facilities that can provide yellow fever vaccinations (Fig. 4A). In addition, since many Japanese clients travel for business, some companies provide malaria prophylaxis in the destination country, and such cases may have been included among clients who did not want to receive malaria prophylaxis. In the future, we believe that this information should be aggregated to obtain a more accurate understanding.

The quality of PTC was assessed previously by confirming the level of knowledge on travel medicine and the simulation of cases with questionnaires [6, 7]. However, in the field of travel medicine, there is a wide variation in individual cases, and it is controversial whether a small number of simulated problems can be used to make a valid quality assessment. Although the frequency of recommended vaccinations according to the guidelines was evaluated by comparing the medical care at multiple institutions in Boston and in an observational study at a single institution [8, 25], the validity of conducting PTCs according to set guidelines also remains controversial. In this study, we assessed the implementation rate of each facility according to the risk of food-borne infectious diseases, mosquito-borne diseases, and rabies in the destination countries and examined indicators that can be used as criteria for evaluating quality. In terms of the implementation of food-borne vaccines and interventions, the GNI, which is a strong predictor of hepatitis A seroprevalence rates [26], was generally below the upper-middle level ($12,535 or less), with nearly 70% receiving diet-related advice and HAV, which could be considered as one criterion. The correlation between the vaccination rate and the incidence of typhoid fever suggested that a typhoid fever vaccination is a good quality indicator in facilities where it is available. For rabies, the rate of education about PEP increased as the risk increased, but the rate of conducting PrEP did not correlate well with the risk; this may have been due to the relatively high cost of PrEP vaccinations and because PrEP is related largely to lifestyle, after travel. Price was also a major factor for the acceptance of vaccinations in Japan, where vaccination is self-financed [27]. Therefore, among long-term travelers, whose vaccination costs are often subsidized by their companies, the rate of PrEP implementation increased with risk (Supplementary Fig. 2 in Additional file 2). Regarding the catch-up immunization rate of measles-containing vaccines, there were large differences in the vaccination rates between facilities, regardless of the vaccination records. As measles outbreaks often occur in young adults [28], it was a good quality indicator that the catch-up immunization rate among clients in their 30s and 40s (the main age reported in PTC) remained high. Although it was difficult to set cutoffs for the quality indicators, the provision of pre-travel counseling by each facility in a way that achieves certain target values for the quality indicators will make it possible to provide more homogeneous and higher quality pre-departure counseling.

### Limitations

This study had three limitations. Although vaccinations were planned, it was unclear whether they were being received. However, a survey conducted by the NCGM on the implementation status of vaccines among clients who first visited in April 2019 showed that over 95% of the planned vaccines were administered (data not shown). Therefore, we believe that the planned vaccines were administered as planned. However, the administration rate was unknown in this study; because whether multiple series could be administered, including vaccinations that were given after travel, is unclear.

Second, the number of enrolled facilities was skewed, with Hospital 2 accounting for more than half of the total. In comparing age, sex, travel purpose, region of travel, and the number of travel regions between Hospital 2 and those of the other facilities, there were no clinically meaningful differences (< 10%) in most of the items. However, Hospital 2, the yellow fever vaccination center, tended to have more travelers going to Africa and South America, more short-term travelers (7–13 days), and fewer people traveling for the business overall (Supplementary Table 6 in Additional file 1). There were limitations related to these.

Third, the socioeconomic statuses of the clients coming for PTC were not known and may not have been equal across the facilities. Although some reports have suggested that cost is not related significantly to vaccination in pre-travel consultations [29], the impact of the cost may vary depending on the country in which the vaccination takes place [27, 30]. The socioeconomic factors should ideally be collected when comparing vaccination rates at different facilities. However, socioeconomic status is not information that is obtained routinely during PTCs, so it may be difficult to obtain this information routinely. It is recommended that this information will be clarified in future studies. However, it can be predicted that there are not too many socioeconomically disadvantaged patients in Japan, given that pre-travel consultations are self-funded and not mandatory for travel, except for YFV.

## Conclusions

The real-world data of PTCs in Japan were obtained from registry-based data and, compared to those of other countries, showed more long-term travelers who traveled for business purposes. The percentage of travelers leaving Japan for low-income countries did not change significantly; however, there were more travelers to the Asian region.

Quality indicators for PTC included: explanations of PEP in high rabies risk countries, HAV rates in low GNI countries, vaccination rates in typhoid risk areas, prescription rates in malaria risk areas, explanations of mosquito control measures in dengue risk countries, and measles vaccination rates in those in their 30s and 40s. Vaccination rates were considered a possible indicator of the quality of care.

## Supplementary Information

**Additional file 1: Supplementary Data: Material and Methods. Supplementary Table 1** Distribution by cooperating facilities. **Supplementary Table 2** Approved and unapproved vaccines in the Japanese government. **Supplementary Table 3** Number of registered cases and age groups per hospital. **Supplementary Table 4** Vaccines and prescriptions by region of travel. **Supplementary Table 5** Acceptance rates of malaria prophylaxis among travelers to high-risk countries (over 10 confirmed cases per 1000 populations) by country. **Supplementary Table 6** Differences in traveler characteristics between Hospital 2 and other hospitals

**Additional file 2: Supplementary Fig. 1** Country classification by income level and hepatitis A vaccinations administered during pre-travel consultation by each collaborated hospital, stratified by the purpose of travel (business, tourism, and others). **Supplementary Fig. 2** Risk classification of rabies and vaccinations administered during pre-travel consultations by each collaborated hospital, stratified by travel duration (181 days or less and more than 181 days). **Supplementary Fig. 3** Risk classification of typhoid fever and vaccinations administered during pre-travel consultations by each collaborated hospital, stratified by the purpose of travel (business, tourism, and others). **Supplementary Fig. 4** Risk classification of falciparum malaria and prophylaxis administered during pre-travel consultations by each collaborated hospital, stratified by the purpose of travel (business, tourism, and others)

## Data Availability

Not applicable.

## Abbreviations

PTC: pre-travel consultations
J-PRECOR: Japan Pretravel Consultation Registry
YFV: yellow fever vaccine
IRB: institutional review board
IQR: interquartile ranges
HAV: hepatitis A vaccination
PrEP: pre-exposure prophylaxis
PEP: post-exposure prophylaxis
GNI: gross national income
