# Supplementary material for: Characteristics and potential quality indicators for evaluating pre-travel consultations in Japan hospitals: the Japan Pretravel consultation registry (J-PRECOR)
Source: Trop Dis Travel Med Vaccines. 2022 Feb 1;8:6. doi: 10.1186/s40794-021-00160-4 (PMC8805374; doi:10.1186/s40794-021-00160-4)
Supplement: Supplementary file 1 — Additional file 1: Supplementary Data: Material and Methods. Supplementary Table 1 Distribution by cooperating facilities. Supplementary Table 2 Approved and unapproved vaccines in the Japanese government. Supplementary Table 3 Number of registered cases and age groups per hospital. Supplementary Table 4 Vaccines and prescriptions by region of travel. Supplementary Table 5 Acceptance rates of malaria prophylaxis among travelers to high-risk countries (over 10 confirmed cases per 1000 populations) by country. Supplementary Table 6 Differences in traveler characteristics between Hospital 2 and other hospitals [file 40794_2021_160_MOESM1_ESM.docx]

**Supplementary Data: Material and Methods**

*Demographic and medical information*

**Sex:** male or female

**Age:** years

**Date of initial consultation:** YYYY/MM/DD

**Departure date:** YYYY/MM/DD, if only the month of travel has been decided, use "YYYY/MM/01": if the beginning, middle, and end of the month have been decided, use "YYYY/MM/05", "YYYY/MM/15", and "YYYY/MM/25", respectively. If the date of departure or return has not been decided, write "undecided".

**Return date:** If only the month of travel has been decided, use "YYYY/MM/01": if the beginning, middle, and end of the month have been decided, use "YYYY/MM/05", "YYYY/MM/15", and "YYYY/MM/25", respectively. If the period of travel is fixed, only the period of travel should be indicated. If the date of departure or return has not been decided, write "undecided".

**Purpose (multiple choice):** Tourism, business, accompanying their family members, volunteer, study, visiting friends and relatives, and others

**Destinations (multiple choice):** Multiple countries can be selected. Transit countries can be entered, but they are not counted in this report.

**Vaccines and medications required by the clients:** Select vaccines and prescriptions that the client has requested prior to the pre-travel consultation.

**Past medical history:** Include history related to vaccines such as measles, rubella, mumps, varicella, and hepatitis B, as well as history of immunodeficiency, severe allergies, or other conditions that may affect vaccination.

**Past immunization record:** The type of vaccines, number of vaccinations, and year of the final dose serological test

**Prescriptions:** Medications including malaria prophylaxis and altitude sickness prophylaxis.

**Vaccinations:** Primary series vaccines or booster vaccines

**Specific advice:** Rabies post-exposure prophylaxis, mosquito control (how to use mosquito repellants such as N,N-diethyl-meta-toluamide [DEET]), freshwater exposure avoidance, precautions on eating and drinking water; precautions on traffic accidents and altitude sickness; prevention of sexually transmitted diseases; purchase of overseas travel insurance; and/or others.

*Evaluating the quality of pre-travel consultations*

The implementation rate of advice or intervention was calculated for facilities that had more than 10 clients in the targeted risk category.

For food-borne diseases

- The gross national income (GNI) category and hepatitis A vaccine percentage: the percentage of vaccine planning was calculated by excluding clients with histories of one series of vaccinations.
- GNI category and the proportion of points to note about eating and drinking.
- Typhoid fever risk category and typhoid vaccine percentage: the percentage of vaccine planning was calculated by excluding clients with the histories of one series of vaccinations.

For animal exposure

- Rabies risk category and advice about rabies post-exposure prophylaxis percentage rabies risk category and rabies pre-exposure prophylaxis percentage: the percentage of vaccine planning was calculated by excluding clients with a histories of one series of rabies pre-exposure prophylaxis.

For mosquito-borne diseases

- *Plasmodium falciparum* malaria risk category and prescribing malaria prophylaxis percentage: for malaria prophylaxis, the data were calculated only for travels within two months.
- Dengue fever risk category and advice about using mosquito repellant percentage.

Catch-up immunization

- Catch-up measles-containing vaccine by age group: the percentage of vaccine planning was calculated by excluding antibody test positive clients. Two doses of vaccine shall be considered as completion of catch-up immunization.

*The country names (ISO 3166-1 codes, Alpha-3 code) included in each risk category*

**GNI**

Country income was categorized into low, lower-middle, upper-middle, and high according to the gross national income (GNI) published by the World Bank.

Countries with a GNI of $12,536 or more: OMN, ARE, SAU, PAN, KOR, ATG, AUS, BHS, BRB, BRN, CHL, KNA, PLW, TTO, ABW, CYM, NCL, PRI, PYF, TCA, TWN, VGB, VIR, FRA, HRV, JPN, SGP, SYC, URY, BMU, HKG, MAC, MNP, AND, AUT, BEL, CAN, CHE, CYP, CZE, DEU, DNK, ESP, EST, FIN, GBR, GRC, IRL, ISL, ISR, ITA, LUX, MCO, MLT, NLD, NOR, NZL, POL, PRT, SMR, SVK, SVN, SWE, USA, BHR, HUN, KWT, LTU, LVA, QAT, CUW, FRO, GIB, GRL, GUM, IMN, LIE, MAF, SXM
Countries with a GNI of between $4,046 and $12,535: PRY, LKA, ARG, ARM, TUR, AZE, DZA, GEO, IRQ, KAZ, TKM, BLZ, CRI, CHN, MYS, GTM, DOM, MEX, IRN, BRA, COL, SUR, THA, PER, ECU, BWA, ZAF, GUY, VEN, NAM, GAB, GNQ, DMA, FJI, GRD, JAM, LCA, MDV, TON, VCT, CUB, ASM, MHL, MUS, NRU, TUV, WSM, SRB, ALB, BGR, BIH, BLR, LBN, LBY, MKD, ROU, RUS, KXX, MNE
Lower-middle countries with a GNI of between $1,036 and $4,045: EGY, UZB, KGZ, MAR, SLV, BTN, TLS, HND, PHL, VNM, CPV, NIC, BOL, IDN, BGD, MMR, PAK, IND, COM, VUT, SWZ, LAO, SDN, DJI, KHM, STP, KEN, SEN, ZWE, MRT, PNG, SLB, COG, ZMB, AGO, GHA, CMR, NGA, CIV, FSM, KIR, JOR, MDA, UKR, MNG, TUN, LSO
Countries with a GNI of $1,035 or less: SYR, TJK, NPL, PRK, HTI, YEM, SOM, ERI, ETH, AFG, MDG, GNB, TZA, TCD, GMB, BDI, MWI, SSD, BFA, UGA, RWA, BEN, MLI, TGO, CAF, COD, GIN, LBR, MOZ, NER, SLE

No data on the reference for the following countries are available: LTE, EAP, EAS, IBD, LAC, LCN, MEA, MNA, SAS, TEA, TLA, TMN, TSA, UMC, ARB, EAR, LMC, MIC, IBT, LMY, WLD, FCS, HPC, IDA, IDB, IDX, LDC, LIC, PRE, SSA, SSF, TSS, COK, GLP, GUF, MTQ, NIU, AIA, MSR, MYT, REU, TKL, WLF, ATA, CCK, CEB, CHI, CSS, CXR, ECA, ECS, EMU, ESH, EUU, FLK, HIC, INX, NAC, NFK, OED, OSS, PSE, PSS, PST, SGS, SHN, SPM, SST, TEC, UMI, XKX

**Rabies incidence among countries (human rabies per 100,000 persons)**

Countries with an incidence of less than 0.0024: AND, ATG, AUS, AUT, BEL, BHS, BLZ, BRB, BRN, CAN, CHE, CHL, COK, COM, CPV, CRI, CYP, CZE, DEU, DMA, DNK, ECU, ESP, EST, FIN, FJI, FRA, FSM, GBR, GRC, GRD, HRV, IRL, ISL, ISR, ITA, JAM, JPN, KIR, KNA, KOR, LCA, LUX, MCO, MDV, MEX, MHL, MLT, MUS, MYS, NIC, NIU, NLD, NOR, NRU, NZL, PAN, PLW, PNG, POL, PRT, PRY, SGP, SLB, SMR, SRB, STP, SVK, SVN, SWE, SYC, TLS, TON, TTO, TUV, URY, USA, VCT, VUT, WSM

Countries with an incidence between 0.0024 and less than 0.038: ALB, ARE, ARG, ARM, BGR, BHR, BIH, BLR, BOL, BRA, COL, CUB, GTM, GUY, HND, HUN, JOR, KWT, LBN, LBY, LTU, LVA, MDA, MKD, OMN, PER, QAT, ROU, RUS, SAU, SUR, TUR, UKR, VEN

Countries with an incidence between 0.038 and less than 0.19: AZE, BWA, DOM, DZA, EGY, GAB, GEO, IDN, IRN, IRQ, KAZ, LKA, MNG, NAM, PHL, PRK, SLV, SYR, THA, TKM, TUN, UZB, ZAF

Countries with an incidence between 0.19 and less than 0.6: BGD, CHN, COG, GHA, GNQ, KGZ, LAO, MAR, PAK, SDN, TCD, TJK, UGA, VNM, ZMB

Countries with an incidence between 0.6 and less than 1.5: AGO, CMR, HTI, KEN, MDG, MRT, NGA, RWA, SEN, SWZ, TZA, YEM

Countries with an incidence between 1.5 and less than 3.0: BEN, BTN, CIV, DJI, GMB, IND, KHM, LSO, MLI, SOM, TGO

Countries with an incidence of 3.0 or more: AFG, BDI, BFA, CAF, COD, ERI, ETH, GIN, GNB, LBR, MMR, MOZ, MWI, NER, NPL, SLE, ZWE

No data on the reference are available for the following countries: ABW, AIA, ARB, ASM, ATA, BMU, CCK, CEB, CHI, CSS, CUW, CXR, CYM, EAP, EAR, EAS, ECA, ECS, EMU, ESH, EUU, FCS, FLK, FRO, GIB, GLP, GRL, GUF, GUM, HIC, HKG, HPC, IBD, IBT, IDA, IDB, IDX, IMN, INX, KXX, LAC, LCN, LDC, LIC, LIE, LMC, LMY, LTE, MAC, MAF, MEA, MIC, MNA, MNE, MNP, MSR, MTQ, MYT, NAC, NCL, NFK, OED, OSS, PRE, PRI, PSE, PSS, PST, PYF, REU, SAS, SGS, SHN, SPM, SSA, SSD, SSF, SST, SXM, TCA, TEA, TEC, TKL, TLA, TMN, TSA, TSS, TWN, UMC, UMI, VGB, VIR, WLD, WLF, XKX

**Typhoid fever (the incidence of typhoid fever per 100,000 persons)**

Countries with an incidence of less than 20: TJK, HTI, UZB, KGZ, SLV, HND, NIC, BOL, SWZ, ZWE, MDA, UKR, MNG, LSO, PRY, ARG, ARM, AZE, GEO, KAZ, TKM, BLZ, CRI, DOM, MEX, BRA, COL, SUR, PER, ECU, BWA, ZAF, GUY, VEN, NAM, DMA, GRD, JAM, LCA, VCT, CUB, SRB, ALB, BGR, BIH, BLR, MKD, ROU, RUS, MNE, PAN, KOR, ATG, AUS, BHS, BRB, BRN, CHL, TTO, PRI, TWN, VIR, FRA, HRV, JPN, SGP, URY, BMU, AND, AUT, BEL, CAN, CHE, CYP, CZE, DEU, DNK, ESP, EST, FIN, GBR, GRC, IRL, ISL, ISR, ITA, LUX, MLT, NLD, NOR, NZL, POL, PRT, SVK, SVN, SWE, USA, HUN, LTU, LVA, GRL

Countries with an incidence between 20 and less than 50: SYR, PRK, GNB, EGY, MAR, JOR, TUN, TUR, DZA, IRQ, GTM, IRN, GAB, GNQ, MUS, LBN, LBY, OMN, ARE, SAU, SYC, BHR, KWT, QAT, GUM

Countries with an incidence between 50 and less than 100: YEM, GMB, CAF, COD, CPV, SDN, STP, SEN, MRT, COG, AGO, CIV, FSM, CHN, FJI, MDV, TON, ASM, MHL, WSM, MNP, PSE

Countries with an incidence of 100 or more: NPL, SOM, ERI, ETH, AFG, MDG, TZA, TCD, BDI, MWI, SSD, BFA, UGA, RWA, BEN, MLI, TGO, GIN, LBR, MOZ, NER, SLE, BTN, TLS, PHL, VNM, IDN, BGD, MMR, PAK, IND, COM, VUT, LAO, DJI, KHM, KEN, PNG, SLB, ZMB, GHA, CMR, NGA, KIR, LKA, MYS, THA

No data on the reference are available for the following countries: NRU, TUV, KXX, KNA, PLW, ABW, CYM, NCL, PYF, TCA, VGB, HKG, MAC, MCO, SMR, CUW, FRO, GIB, IMN, LIE, MAF, SXM, LTE, EAP, EAS, IBD, LAC, LCN, MEA, MNA, SAS, TEA, TLA, TMN, TSA, UMC, ARB, EAR, LMC, MIC, IBT, LMY, WLD, FCS, HPC, IDA, IDB, IDX, LDC, LIC, PRE, SSA, SSF, TSS, COK, GLP, GUF, MTQ, NIU, AIA, MSR, MYT, REU, TKL, WLF, ATA, CCK, CEB, CHI, CSS, CXR, ECA, ECS, EMU, ESH, EUU, FLK, HIC, INX, NAC, NFK, OED, OSS, PSS, PST, SGS, SHN, SPM, SST, TEC, UMI, XKX

***P. Falciparum* malaria (the incidence of falciparum malaria per 100,000 persons)**

These countries have no risk: PRY, LKA, ARG, OMN, EGY, ARE, ARM, TUR, AZE, DZA, GEO, IRQ, KAZ, SYR, TKM, UZB, KGZ, MAR

Countries with an incidence of less than 0.1: BLZ, CRI, SLV, BTN, SAU, CHN, TJK

Countries with an incidence between 0.1 and less than 1.0: MYS, PAN, TLS, GTM, HND, DOM, PHL, VNM, NPL, MEX, CPV, KOR, IRN, PRK, LTE

Countries with an incidence between 1.0 and less than 10: NIC, BOL, BRA, COL, SUR, IDN, THA, BGD, HTI, MMR, PER, PAK, IND, COM, ECU, VUT, BWA, ZAF, SWZ, EAP, EAS, IBD, LAC, LCN, MEA, MNA, SAS, TEA, TLA, TMN, TSA, UMC

Countries with an incidence between 10 and less than 50: GUY, VEN, LAO, SDN, YEM, DJI, KHM, SOM, ERI, ETH, STP, NAM, AFG, ARB, EAR, LMC, MIC

Countries with an incidence between 50 and less than 100: KEN, MDG, SEN, GNB, ZWE, MRT, IBT, LMY, WLD

Countries with an incidence between 100 and less than 250: PNG, SLB, TZA, COG, TCD, ZMB, AGO, GMB, BDI, MWI, SSD, FCS, HPC, IDA, IDB, IDX, LDC, LIC, PRE, SSA, SSF, TSS

Countries with an incidence of 250 or more: BFA, GAB, GHA, GNQ, UGA, CMR, NGA, RWA, BEN, CIV, MLI, TGO, CAF, COD, GIN, LBR, MOZ, NER, SLE

No data on the reference are available for the following countries: ATG, AUS, BHS, BRB, BRN, CHL, COK, DMA, FJI, FSM, GRD, JAM, KNA, LCA, MDV, PLW, TON, TTO, VCT, CUB, ABW, ASM, CYM, GLP, GUF, MTQ, NCL, PRI, PYF, TCA, TWN, VGB, VIR, FRA, HRV, JPN, KIR, MHL, MUS, NIU, NRU, SGP, SYC, TUV, URY, WSM, AIA, BMU, HKG, MAC, MNP, MSR, MYT, REU, TKL, WLF, AND, AUT, BEL, CAN, CHE, CYP, CZE, DEU, DNK, ESP, EST, FIN, GBR, GRC, IRL, ISL, ISR, ITA, LUX, MCO, MLT, NLD, NOR, NZL, POL, PRT, SMR, SRB, SVK, SVN, SWE, USA, ALB, BGR, BHR, BIH, BLR, HUN, JOR, KWT, LBN, LBY, LTU, LVA, MDA, MKD, QAT, ROU, RUS, UKR, MNG, TUN, LSO, ATA, CCK, CEB, CHI, CSS, CUW, CXR, ECA, ECS, EMU, ESH, EUU, FLK, FRO, GIB, GRL, GUM, HIC, IMN, INX, KXX, LIE, MAF, MNE, NAC, NFK, OED, OSS, PSE, PSS, PST, SGS, SHN, SPM, SST, SXM, TEC, UMI, XKX

**Dengue fever**

No or unknown risk (no data on the reference are available for the following countries): AND, AUT, BEL, CAN, CHE, CYP, CZE, DEU, DNK, ESP, EST, FIN, GBR, GRC, IRL, ISL, ISR, ITA, LUX, MCO, MLT, NLD, NOR, NZL, POL, PRT, SMR, SRB, SVK, SVN, SWE, USA, ALB, ARE, ARM, BGR, BHR, BIH, BLR, HUN, JOR, KWT, LBN, LBY, LTU, LVA, MDA, MKD, QAT, ROU, RUS, TUR, UKR, AZE, BWA, DZA, GEO, IRQ, KAZ, MNG, PRK, SYR, TKM, TUN, UZB, ZAF, KGZ, MAR, TJK, MRT, SWZ, LSO, ARB, ATA, CCK, CEB, CHI, CSS, CUW, CXR, EAP, EAR, EAS, ECA, ECS, EMU, ESH, EUU, FCS, FLK, FRO, GIB, GRL, GUM, HIC, HPC, IBD, IBT, IDA, IDB, IDX, IMN, INX, KXX, LAC, LCN, LDC, LIC, LIE, LMC, LMY, LTE, MAF, MEA, MIC, MNA, MNE, NAC, NFK, OED, OSS, PRE, PSE, PSS, PST, SAS, SGS, SHN, SPM, SSA, SSF, SST, SXM, TEA, TEC, TLA, TMN, TSA, TSS, UMC, UMI, WLD, XKX

The risk varies based on the region: MEX, ARG, PER, SAU, CHN, PAK, IND

Sporadic/uncertain: COM, CPV, ECU, FRA, HRV, JPN, KIR, KOR, MHL, MUS, NIU, NRU, SGP, STP, SYC, TUV, URY, VUT, WSM, OMN, EGY, GAB, IRN, NAM, COG, GHA, GNQ, TCD, UGA, ZMB, AGO, CMR, MDG, NGA, RWA, SEN, BEN, CIV, GMB, MLI, TGO, AFG, BDI, CAF, COD, GIN, GNB, LBR, MOZ, MWI, NER, SLE, ZWE, AIA, BMU, HKG, MAC, MNP, MSR, MYT, REU, SSD, TKL, WLF

Frequent/continuous: ATG, AUS, BHS, BLZ, BRB, BRN, CHL, COK, CRI, DMA, FJI, FSM, GRD, JAM, KNA, LCA, MDV, MYS, NIC, PAN, PLW, PNG, PRY, SLB, TLS, TON, TTO, VCT, BOL, BRA, COL, CUB, GTM, GUY, HND, SUR, VEN, DOM, IDN, LKA, PHL, SLV, THA, BGD, LAO, SDN, VNM, HTI, KEN, TZA, YEM, BTN, DJI, KHM, SOM, BFA, ERI, ETH, MMR, NPL, ABW, ASM, CYM, GLP, GUF, MTQ, NCL, PRI, PYF, TCA, TWN, VGB, VIR

Supplementary Table 1. Distribution by cooperating facilities

|  | Annual estimated number of cases for pretravel consultations | Population of prefectures where the hospital is located | Vaccines unapproved by the government of Japan | Yellow fever vaccine | Duration of registration |
| --- | --- | --- | --- | --- | --- |
| Hospital 01 | 501 to 1,000 cases | 8,839,469 | Yes | Yes | Jul 2019 |
| Hospital 02 | over 1,000 cases | 13,515,271 | Yes | Yes | Feb 2018 |
| Hospital 03 | over 1,000 cases | 1,364,316 | Yes | No | Feb 2018 |
| Hospital 04 | 100 to 500 cases | 2,610,353 | No | No | Feb 2018 |
| Hospital 05 | 100 to 500 cases | 5,151,560 | Yes | No | Feb 2018 |
| Hospital 06 | under 100 cases | 573,441 | No | No | Jun 2018 |
| Hospital 07 | 100 to 500 cases | 2,098,804 | Yes | No | Mar 2018 |
| Hospital 08 | 100 to 500 cases | 5,381,733 | Yes | No | Jun 2019 |
| Hospital 09 | 100 to 500 cases | 8,839,469 | Yes | Yes | Feb 2020 |
| Hospital 10 | 501 to 1,000 cases | 3,700,305 | Yes | No | Jul 2018 to Jun 2019 |
| Hospital 11 | under 100 cases | 834,930 | Yes | No | Jun 2019 |
| Hospital 12 | under 100 cases | 5,151,560 | No | Yes | Jun 2019 |
| Hospital 13 | 100 to 500 cases | 5,151,560 | Yes | No | Jul 2019 |
| Hospital 14 | under 100 cases | 963,579 | Yes | No | Aug 2018 to Mar 2019 |
| Hospital 15 | under 100 cases | 2,843,990 | Yes | No | Mar 2020 |
| Hospital 16 | under 100 cases | 1,364,316 | Yes | No | Oct 2019 |
| Hospital 17 | under 100 cases | 1,066,328 | No | No | Mar 2020 |

Supplementary Table 2. Approved and unapproved vaccines in the Japanese government

| Approved vaccines by Japanese government | Unapproved vaccines by Japanese government |
| --- | --- |
| Inactivated hepatitis A without adjuvant | Inactivated hepatitis A with adjuvant |
| Inactivated hepatitis B | Inactivated hepatitis B with new adjuvant |
| 10- or 13-valent pneumococcal conjugate | Hepatitis A and hepatitis B combined |
| 23-valent pneumococcal polysaccharide | Diphtheria-tetanus-acellular pertussis (Tdap) |
| Hemophilus influenzae b | Diphtheria-tetanus (Td) |
| ^*1^Diphtheria-tetanus-acellular pertussis (DTaP) | Rabies (Verorab) |
| Diphtheria-tetanus (DT) | Meningococcal ACWY (other than Menactra) |
| Diphtheria toxoid | Meningococcal C |
| Tetanus toxoid | Meningococcal B |
| DTaP combined with inactivate poliomyelitis | Typhoid fever polysaccharide |
| Inactivate poliomyelitis | Typhoid fever conjugate |
| BCG | Tick-borne encephalitis |
| Inactivated Japanese encephalitis | Measles, mumps, and rubella |
| Measles and rubella | Measles, mumps, rubella, and varicella |
| Measles | Live attenuated influenza |
| Rubella | Inactivated oral cholera |
| Mumps | Live attenuated hepatitis A |
| Live attenuated varicella/zoster | Live attenuated Japanese encephalitis |
| ^*2^Inactivated zoster | Dengue |
| ^*3^Rabies (Rabipur) |  |
| Meningococcal ACWY (Menactra) |  |
| Inactivated influenza |  |
| Yellow fever |  |
| ^*4^9-, 4-, or 2-valent Human papillomavirus |  |

Supplementary Table 3. Number of registered cases and age groups per hospital

| Hospitals | All | Age 0 to 15 years | Age 16 to 64 years | Age 65 years or over |
| --- | --- | --- | --- | --- |
| Number of cases | 9,700 | 880 | 8,271 | 549 |
| Hospital 1 | 853 (8.8) | 18 (2) | 788 (9.5) | 47 (8.6) |
| Hospital 2 | 5,591 (57.6) | 463 (52.6) | 4,723 (57.1) | 405 (73.8) |
| Hospital 3 | 1,030 (10.6) | 201 (22.8) | 788 (9.5) | 41 (7.5) |
| Hospital 4 | 308 (3.2) | 15 (1.7) | 291 (3.5) | 2 (0.4) |
| Hospital 5 | 962 (9.9) | 132 (15) | 807 (9.8) | 23 (4.2) |
| Hospital 6 | 57 (0.6) | 2 (0.2) | 54 (0.7) | 1 (0.2) |
| Hospital 7 | 303 (3.1) | 22 (2.5) | 266 (3.2) | 15 (2.7) |
| Hospital 8 | 47 (0.5) | 1 (0.1) | 45 (0.5) | 1 (0.2) |
| Hospital 9 | 93 (1) | 5 (0.6) | 82 (1) | 6 (1.1) |
| Hospital 10 | 182 (1.9) | 0 (0) | 179 (2.2) | 3 (0.5) |
| Hospital 11 | 40 (0.4) | 2 (0.2) | 36 (0.4) | 2 (0.4) |
| Hospital 12 | 85 (0.9) | 0 (0) | 83 (1) | 2 (0.4) |
| Hospital 13 | 24 (0.2) | 4 (0.5) | 19 (0.2) | 1 (0.2) |
| Hospital 14 | 56 (0.6) | 8 (0.9) | 48 (0.6) | 0 (0) |
| Hospital 15 | 37 (0.4) | 3 (0.3) | 34 (0.4) | 0 (0) |
| Hospital 16 | 13 (0.1) | 1 (0.1) | 12 (0.1) | 0 (0) |
| Hospital 17 | 19 (0.2) | 3 (0.3) | 16 (0.2) | 0 (0) |

Supplementary Table 4. Vaccines and prescriptions by region of travel

|  | All | Asia | Africa | South America | Others | Multiple regions |
| --- | --- | --- | --- | --- | --- | --- |
| N (%) | 9,700 | 4,008 | 2,593 | 1,809 | 1,660 | 834 |
| Vaccines and prescriptions that the participants wanted themselves | | | | | | |
| Hepatitis A vaccine | 3,946 (40.7) | 2,507 (62.5) | 778 (30.0) | 497 (27.5) | 311 (18.7) | 281 (33.7) |
| Hepatitis B vaccine | 2,562 (26.4) | 1,667 (41.6) | 323 (12.5) | 239 (13.2) | 399 (24.0) | 133 (15.9) |
| Rabies vaccine | 2,804 (28.9) | 1,925 (48.0) | 447 (17.2) | 318 (17.6) | 224 (13.5) | 170 (20.4) |
| Vaccines containing tetanus toxoid | 3,017 (31.1) | 1,732 (43.2) | 532 (20.5) | 351 (19.4) | 507 (30.5) | 194 (23.3) |
| Tdap | 151 (1.6) | 17 (0.4) | 3 (0.1) | 2 (0.1) | 131 (7.9) | 5 (0.6) |
| DTaP | 471 (4.9) | 212 (5.3) | 113 (4.4) | 64 (3.5) | 96 (5.8) | 23 (2.8) |
| Typhoid fever vaccine | 1,513 (15.6) | 1,035 (25.8) | 318 (12.3) | 154 (8.5) | 61 (3.7) | 101 (12.1) |
| Japanese encephalitis vaccine | 1,231 (12.7) | 1,138 (28.4) | 49 (1.9) | 33 (1.8) | 38 (2.3) | 37 (4.4) |
| Meningococcal ACWY vaccine | 463 (4.8) | 49 (1.2) | 144 (5.6) | 7 (0.4) | 275 (16.6) | 29 (3.5) |
| Meningococcal B vaccine | 8 (0.1) | 0 (0) | 1 (0) | 2 (0.1) | 5 (0.3) | 1 (0.1) |
| Vaccines containing measles | 772 (8.0) | 443 (11.1) | 63 (2.4) | 43 (2.4) | 240 (14.5) | 30 (3.6) |
| Vaccines containing rubella | 682 (7.0) | 380 (9.5) | 55 (2.1) | 39 (2.2) | 217 (13.1) | 23 (2.8) |
| Yellow fever vaccine | 3,014 (31.1) | 185 (4.6) | 1,662 (64.1) | 1,254 (69.3) | 128 (7.7) | 484 (58.0) |
| Prophylaxis for acute altitude sickness | 338 (3.5) | 40 (1.0) | 63 (2.4) | 261 (14.4) | 10 (0.6) | 67 (8.0) |
| Prophylaxis for malaria | 1,146 (11.8) | 204 (5.1) | 830 (32.0) | 168 (9.3) | 34 (2.0) | 167 (20.0) |
| Vaccines and prescriptions actually given after pre-travel consultation | | | | | | |
| Hepatitis A vaccine | 5,655 (58.3) | 3,253 (81.2) | 1,293 (49.9) | 828 (45.8) | 518 (31.2) | 469 (56.2) |
| Hepatitis A vaccine -Approved | 2,802 (28.9) | 1,774 (44.3) | 584 (22.5) | 353 (19.5) | 224 (13.5) | 206 (24.7) |
| Hepatitis A vaccine -Not approved* | 2,858 (29.5) | 1,484 (37) | 709 (27.3) | 475 (26.3) | 294 (17.7) | 263 (31.5) |
| Hepatitis B vaccine | 2,961 (30.5) | 1,909 (47.6) | 328 (12.6) | 257 (14.2) | 546 (32.9) | 139 (16.7) |
| Hepatitis B vaccine -Approved | 2,485 (25.6) | 1,519 (37.9) | 304 (11.7) | 234 (12.9) | 505 (30.4) | 126 (15.1) |
| Hepatitis B vaccine -Not approved* | 482 (5) | 395 (9.9) | 24 (0.9) | 23 (1.3) | 42 (2.5) | 13 (1.6) |
| Rabies vaccine | 3,209 (33.1) | 2,264 (56.5) | 515 (19.9) | 336 (18.6) | 219 (13.2) | 174 (20.9) |
| Rabies vaccine -Approved | 624 (6.4) | 491 (12.3) | 69 (2.7) | 46 (2.5) | 39 (2.3) | 24 (2.9) |
| Rabies vaccine -Not approved | 2,338 (24.1) | 1,595 (39.8) | 402 (15.5) | 256 (14.2) | 166 (10) | 129 (15.5) |
| Vaccines containing tetanus toxoid | 4,625 (47.7) | 2,321 (57.9) | 934 (36) | 621 (34.3) | 944 (56.9) | 356 (42.7) |
| Tetanus toxoid | 1,733 (17.9) | 1,053 (26.3) | 372 (14.3) | 263 (14.5) | 140 (8.4) | 161 (19.3) |
| DTaP | 2,388 (24.6) | 1,214 (30.3) | 598 (23.1) | 376 (20.8) | 307 (18.5) | 198 (23.7) |
| Tdap | 597 (6.2) | 95 (2.4) | 20 (0.8) | 14 (0.8) | 474 (28.6) | 14 (1.7) |
| Others | 107 (1.1) | 39 (1) | 10 (0.4) | 8 (0.4) | 51 (3.1) | 2 (0.2) |
| Typhoid fever vaccine | 2,468 (25.4) | 1,624 (40.5) | 601 (23.2) | 285 (15.8) | 88 (5.3) | 203 (24.3) |
| Japanese encephalitis | 1,745 (18) | 1,598 (39.9) | 70 (2.7) | 56 (3.1) | 81 (4.9) | 61 (7.3) |
| Meningococcus ACWY | 772 (8) | 76 (1.9) | 214 (8.3) | 20 (1.1) | 481 (29) | 43 (5.2) |
| Meningococcus B | 32 (0.3) | 2 (0) | 3 (0.1) | 3 (0.2) | 24 (1.4) | 1 (0.1) |
| Vaccines containing measles | 2,012 (20.7) | 1,050 (26.2) | 307 (11.8) | 215 (11.9) | 506 (30.5) | 130 (15.6) |
| Vaccines containing rubella | 2,006 (20.7) | 1,039 (25.9) | 307 (11.8) | 217 (12) | 510 (30.7) | 132 (15.8) |
| Yellow fever vaccine | 3,559 (36.7) | 215 (5.4) | 2,005 (77.3) | 1,466 (81) | 130 (7.8) | 590 (70.7) |
|  |  |  |  |  |  |  |
| Prophylaxis for acute altitude sickness | 370 (3.8) | 52 (1.3) | 60 (2.3) | 296 (16.4) | 13 (0.8) | 83 (10.0) |
| Prophylaxis for malaria | 1,252 (12.9) | 132 (3.3) | 1,071 (41.3) | 118 (6.5) | 29 (1.7) | 232 (27.8) |
| Atovaquone-proguanil | 935 (9.6) | 87 (2.2) | 807 (31.1) | 84 (4.6) | 22 (1.3) | 170 (20.4) |
| Doxycycline | 240 (2.5) | 37 (0.9) | 196 (7.6) | 19 (1.1) | 6 (0.4) | 44 (5.3) |
| Mefloquine | 77 (0.8) | 8 (0.2) | 68 (2.6) | 15 (0.8) | 1 (0.1) | 18 (2.2) |
| *Including Hepatitis A and B combined vaccine | | | | | | |

Supplementary Table 5. Acceptance rates of malaria prophylaxis among travelers to high-risk countries (over 10 confirmed cases per 1,000 populations) by country

| Countries | Recommend cases | Prescribed cases | Rate |
| --- | --- | --- | --- |
| Togo | 19 | 16 | 84.2 |
| Papua New Guinea | 12 | 10 | 83.3 |
| Zimbabwe | 56 | 46 | 82.1 |
| Gabon | 11 | 9 | 81.8 |
| Sierra Leone | 16 | 13 | 81.2 |
| Benin | 44 | 35 | 79.5 |
| Malawi | 33 | 26 | 78.8 |
| Nigeria | 88 | 68 | 77.3 |
| Zambia | 78 | 59 | 75.6 |
| Republic of the Congo | 15 | 11 | 73.3 |
| Senegal | 71 | 52 | 73.2 |
| Ghana | 192 | 137 | 71.4 |
| Tanzania | 252 | 173 | 68.7 |
| Burkina Faso | 19 | 13 | 68.4 |
| Uganda | 220 | 150 | 68.2 |
| Rwanda | 95 | 60 | 63.2 |
| Ivory Coast | 84 | 52 | 61.9 |
| Madagascar | 31 | 19 | 61.3 |
| Angola | 18 | 11 | 61.1 |
| **Kenya** | 469 | 279 | **59.5** |
| **Democratic Republic of Congo** | 37 | 21 | **56.8** |
| **Mozambique** | 26 | 14 | **53.8** |
| **Cameroon** | 17 | 8 | **47.1** |
| **Guinea** | 19 | 8 | **42.1** |

Supplementary Table 6. Differences in traveler characteristics between Hospital 2 and the other hospitals

|  | Hospital 2 | Other hospitals | Percentage difference |
| --- | --- | --- | --- |
| Males, n (%) | 3,172 (56.8) | 2,634 (64.2) | 7.4 |
| Age, median (IQR) | 31 (21–45) | 33 (22–44) | NA |
| Duration of travel, n (%) |  |  |  |
| <7 d | 405 (7.3) | 270 (6.6) | -0.7 |
| 7–13 d | 1,660 (29.7) | 612 (14.9) | -14.8 |
| 14–27 d | 961 (17.2) | 507 (12.4) | -4.9 |
| 28–55 d | 467 (8.4) | 344 (8.4) | 0.1 |
| 56–181 d | 399 (7.2) | 275 (6.7) | -0.5 |
| >181 d | 1,475 (26.4) | 1,815 (44.2) | 17.8 |
| Unknown/undetermined | 224 (4.1) | 286 (7.0) | 3.0 |
| Destination of travel, n (%) |  |  |  |
| Asia | 1,623 (29.1) | 2,385 (58.1) | 29.1 |
| Africa | 1,976 (35.4) | 617 (15.1) | -20.4 |
| SA | 1,351 (24.2) | 458 (11.2) | -13.1 |
| NA | 748 (13.4) | 510 (12.5) | -1.0 |
| Europe | 335 (6.0) | 219 (5.4) | -0.7 |
| Middle East | 306 (5.5) | 108 (2.7) | -2.9 |
| Oceania | 80 (1.5) | 71 (1.8) | 0.3 |
| Others | 52 (1.0) | 54 (1.4) | 0.4 |
| Number of countries for visiting | 1 (1–1) | 1 (1–1) | NA |
| Purpose of travel, n (%) |  |  |  |
| Tourism tour | 455 (8.2) | 185 (4.6) | -3.7 |
| Tourism others | 1,300 (23.3) | 610 (14.9) | -8.5 |
| Business | 2,022 (36.2) | 1,908 (46.5) | 10.3 |
| Accompanying | 530 (9.5) | 668 (16.3) | 6.8 |
| Migration | 15 (0.3) | 11 (0.3) | -0.1 |
| Study | 882 (15.8) | 448 (11.0) | -4.9 |
| Volunteer | 325 (5.9) | 147 (3.6) | -2.3 |
| VFR | 111 (2.0) | 21 (0.6) | -1.5 |
| Others | 53 (1.0) | 161 (4.0) | 3.0 |

IQR, interquartile range; SA, South Africa; NA, not applicable; VFR, visiting friends and relatives
