# Supplementary material for: Characteristics and potential quality indicators for evaluating pre-travel consultations in Japan hospitals: the Japan Pretravel consultation registry (J-PRECOR)
Source: Trop Dis Travel Med Vaccines. 2022 Feb 1;8:6. doi: 10.1186/s40794-021-00160-4 (PMC8805374; doi:10.1186/s40794-021-00160-4)
Supplement: Supplementary file 2 — Additional file 2: Supplementary Fig. 1 Country classification by income level and hepatitis A vaccinations administered during pre-travel consultation by each collaborated hospital, stratified by the purpose of travel (business, tourism, and others). Supplementary Fig. 2 Risk classification of rabies and vaccinations administered during pre-travel consultations by each collaborated hospital, stratified by travel duration (181 days or less and more than 181 days). Supplementary Fig. 3 Risk classification of typhoid fever and vaccinations administered during pre-travel consultations by each collaborated hospital, stratified by the purpose of travel (business, tourism, and others). Supplementary Fig. 4 Risk classification of falciparum malaria and prophylaxis administered during pre-travel consultations by each collaborated hospital, stratified by the purpose of travel (business, tourism, and others) [file 40794_2021_160_MOESM2_ESM.pdf]

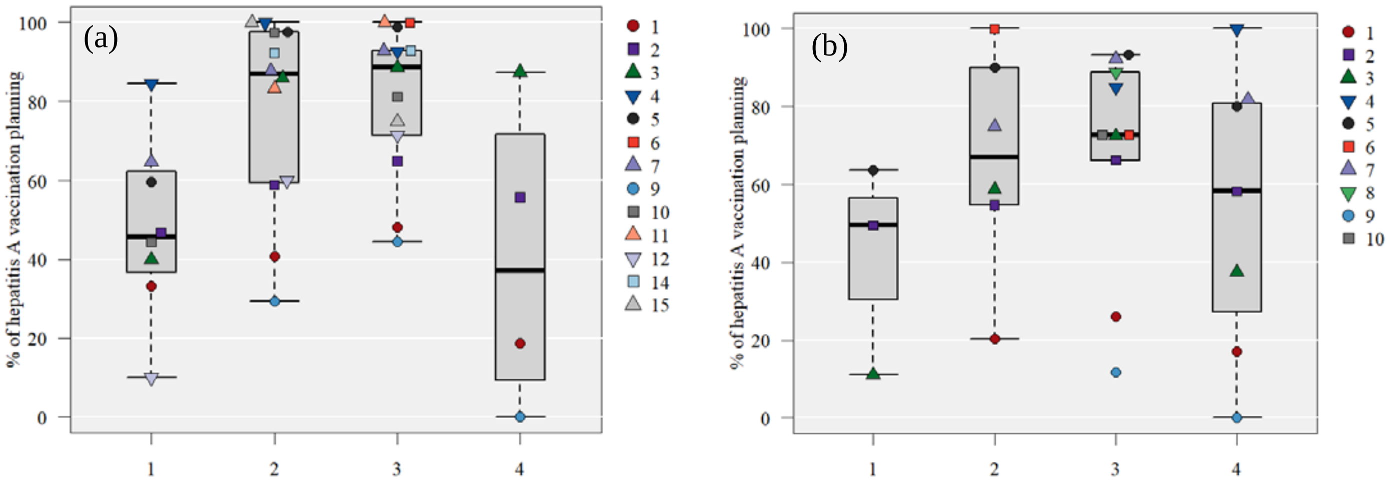

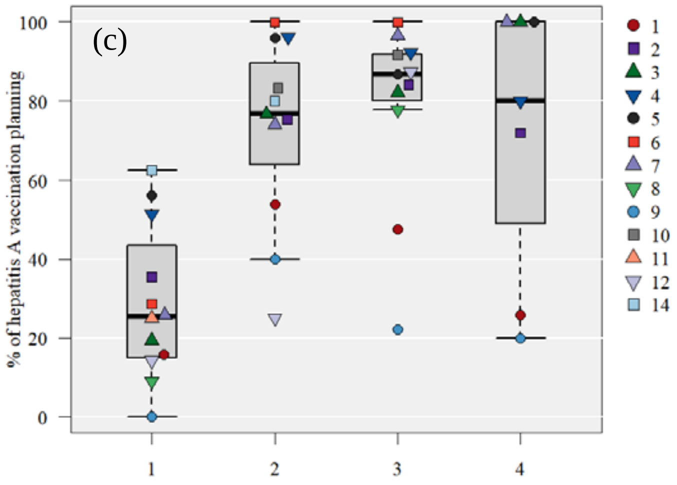

Supplementary Figure 1. Country classification by income level and hepatitis A vaccinations administered during pre-travel consultation by each collaborated hospital, stratified by purpose of travel (business, tourism, and others)

(a) Purpose of travel: business

(b) Purpose of travel: tourism

(c) Purpose of travel: others

The risk categories, in order, from 1 to 4, are "high GNI: $12,536 or more"; "upper-middle GNI: $4,046 and $12,535"; "lower-middle GNI: $1,036 and $4,045"; and "low GNI: $1,035 or less."

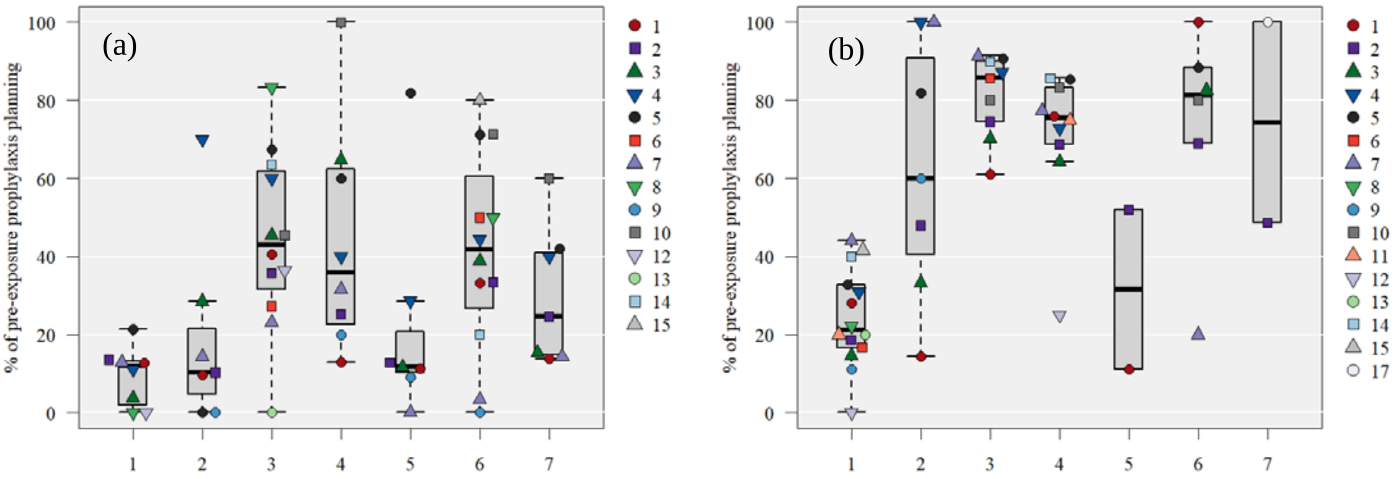

Supplementary Figure 2. Risk classification of rabies and vaccinations administered during pre-travel consultations by each collaborated hospital, stratified by travel duration (181 days or less and more than 181 days)

(a) Travel duration:181 days or less

(b) Travel duration: more than 181 days

The risk categories, in order, from one to seven, are death rates due to human rabies per capita (per 100,000 persons): "less than 0.0024"; "0.0024 to less than 0.038"; "0.038 to less than 0.19"; 0.19 to less than 0.6"; "0.6 to less than 1.5"; "1.5 to less than 3.0"; and "3.0 or more."

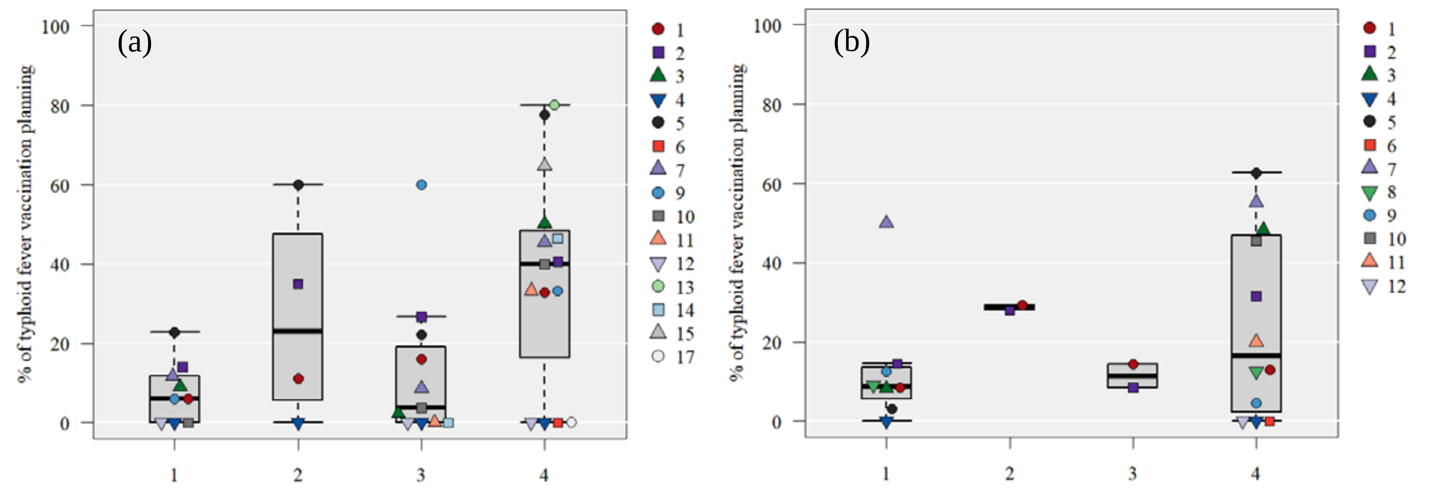

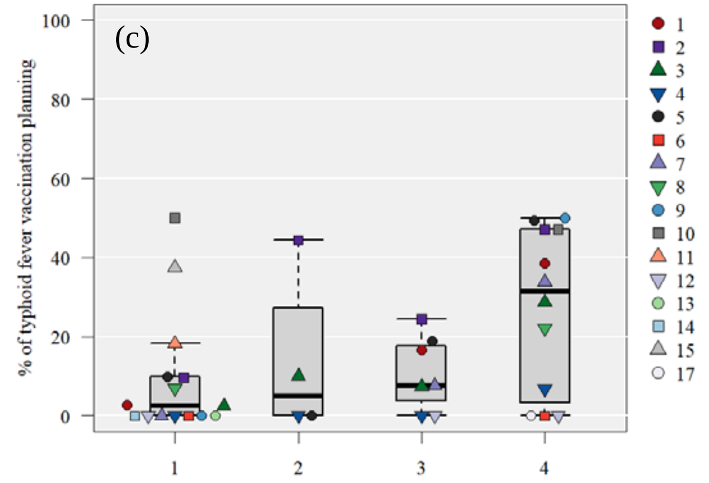

Supplementary Figure 3. Risk classification of typhoid fever and vaccinations administered during pre-travel consultations by each collaborated hospital, stratified by purpose of travel (business, tourism, and others)

(a) Purpose of travel: business
(b) Purpose of travel: tourism
(c) Purpose of travel: others

The risk categories, in order, from one to four, are the incidence of typhoid fever per 100,000 persons: "less than 20"; "20 to less than 50”; "50 to less than 100”; and "100 or more.”

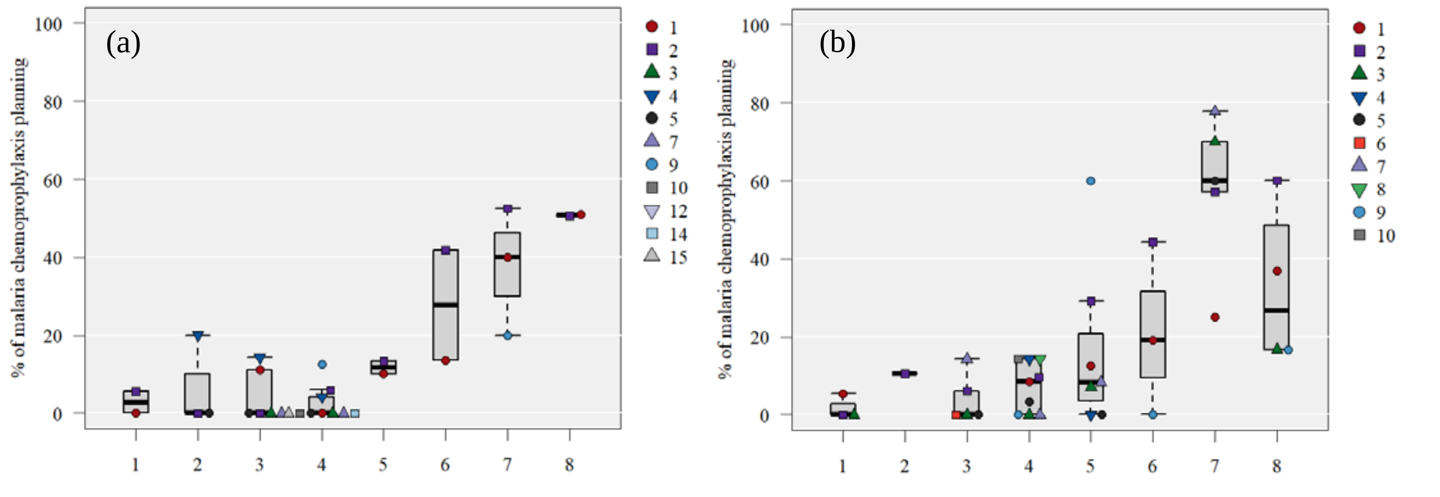

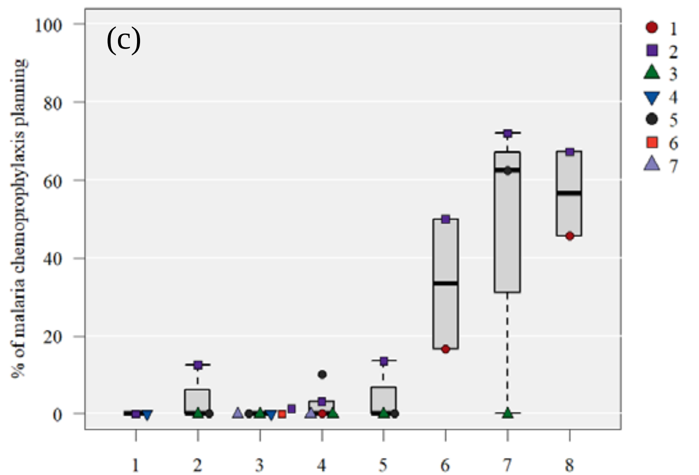

Supplementary Figure 4. Risk classification of falciparum malaria and prophylaxis administered during pre-travel consultations by each collaborated hospital, stratified by purpose of travel (business, tourism, and others)

(a) Purpose of travel: business
(b) Purpose of travel: tourism
(c) Purpose of travel: others

The risk categories, in order, from one to eight, are the incidence of falciparum malaria per 1,000 persons: “no risk”; "less than 0.1"; "0.1 to less than 1.0”; "1.0 to less than 10”; “10 to less than 50”; “50 to less than 100”; “100 to less than 250”; and "250 or more.”
